# NME/NM23/NDPK and Histidine Phosphorylation

**DOI:** 10.3390/ijms21165848

**Published:** 2020-08-14

**Authors:** Kevin Adam, Jia Ning, Jeffrey Reina, Tony Hunter

**Affiliations:** Molecular and Cell Biology Laboratory, Salk Institute for Biological Studies, La Jolla, CA 92037, USA; kadam@salk.edu (K.A.); jning@salk.edu (J.N.); jreina@salk.edu (J.R.)

**Keywords:** NME, histidine kinase, phosphorylation

## Abstract

The NME (Non-metastatic) family members, also known as NDPKs (nucleoside diphosphate kinases), were originally identified and studied for their nucleoside diphosphate kinase activities. This family of kinases is extremely well conserved through evolution, being found in prokaryotes and eukaryotes, but also diverges enough to create a range of complexity, with homologous members having distinct functions in cells. In addition to nucleoside diphosphate kinase activity, some family members are reported to possess protein-histidine kinase activity, which, because of the lability of phosphohistidine, has been difficult to study due to the experimental challenges and lack of molecular tools. However, over the past few years, new methods to investigate this unstable modification and histidine kinase activity have been reported and scientific interest in this area is growing rapidly. This review presents a global overview of our current knowledge of the NME family and histidine phosphorylation, highlighting the underappreciated protein-histidine kinase activity of NME family members, specifically in human cells. In parallel, information about the structural and functional aspects of the NME family, and the knowns and unknowns of histidine kinase involvement in cell signaling are summarized.

## 1. Histidine Kinase in Mammals: from Myth to Reality?

Ubiquitous nucleoside diphosphate kinases (NDPKs) were identified early in the 1950s based on their ability to catalyze the transfer of a phosphate from nucleoside triphosphates (NTPs) to nucleoside diphosphates (NDPs) in yeast [1] and pigeon heart muscle [2]. A few years later, the existence of phosphorylated histidine (phosphohistidine, pHis) residues in proteins was discovered [3], and then pHis was found to be present on NDPK proteins extracted from alkaline lysates of Jerusalem artichoke mitochondria, bovine liver, and baker’s yeast [4,5,6,7].

This review will discuss the complexity and polyvalence of NDPK activities and the challenges faced in working on this unstable post-translational modification, and outline the progress that has been made in the past few years in understanding how protein-histidine phosphorylation is catalyzed by NME family members and used to regulate cellular functions [8]. 

### 1.1. Complexity of NME Genes and Functions

**Figure 1:**
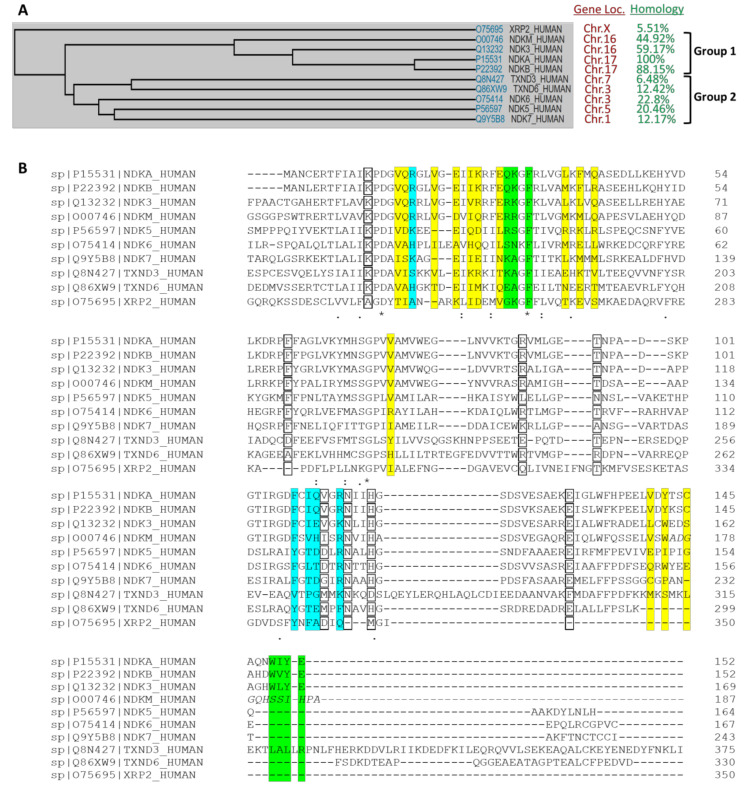
Global comparison of human NME family members. NME1 to 10 correspond respectively to NDKA, NDKB, NDK3, NDKM, NDK5, NDK6, NDK7, TXND3, TXND6 and XRP2. (**A**) Evolutionary tree from the sequence alignment of the 10 NME proteins in humans using the Uniprot database. Their respective gene localization is provided, as well as the protein sequence identity based on NME1 (NDKA) as reference. (**B**) Catalytic site in protein sequence alignment of human NME1 to NME10. Uniprot IDs of each gene encoding human NME1-10 are shown at the start of each line. Residues lying in the active site are highlighted with boxes. Residues that are responsible for forming large interface, small interface 1, and small interface 2, are highlighted in yellow, cyan, and green, respectively. Residues identical in all aligned NMEs are labeled by an asterisk (*), whereas a colon (:) and a period (.) indicate strongly similar and weakly similar sequences, respectively. Several amino acids missing in the NME4 crystal structure (Protein Data Bank (PDB) ID: 1EHW) due to the weak density are shown in italics.

NDPK enzymes are expressed from *NME* (for non-metastatic) genes, also called *NM23* (for non-metastatic protein 23) genes, and are highly conserved through evolution with multiple synonyms gene names in different organisms, including *AWD, NDK, YNK, Gip17/Guk, NmeGp1sd* [9]. This multitude of names illustrates the presence of orthologous and paralogous members. For example, in humans the NME family has 10 different members divided into two main groups [10]. A whole protein sequence comparison is provided in Figure 1. The first group includes the well-conserved NME1-4 (45–88% overall identity to NME1, 58–88% identity in terms of NDPK domain only alignment [10] that are catalytically active. The second group includes NME5-9 that are far more divergent (6–23% overall identity to NME1, 22% to 44% if only NDPK domain is considered [10]). There is no consistent evidence to confirm the catalytic activities of group II members. Two papers reported no NDPK activity for NME7 [11,12] although one study found NME7 possesses autophosphorylation activity. For NME6, contradictory results have been reported both for autophosphorylation and NDPK activities [11,13]. NME10 (XRP2) is separate from these two groups having a different evolutionary history [14].

**Figure 2:**
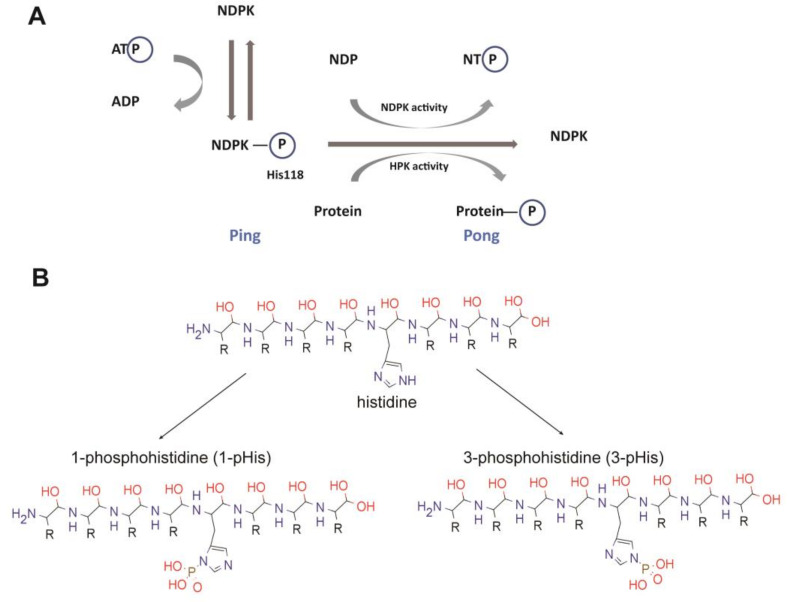
Catalytic mechanism of NDPKs. (**A**) The process of phosphotransfer through NDPK catalysis. The transferred phosphate group is highlighted by blue circles. (**B**) The intermediate states of phosphotransfer through pHis (1-pHis or 3-pHis).

The sequence alignment reveals common features, particularly in the catalytic domain, but distinct features in loops and the N- and C-termini. As mentioned above, NME kinases were first associated with a nucleoside diphosphate kinase (NDPK) activity that catalyzed the transfer of the γ-phosphate from nucleoside triphosphates through a phosphohistidine enzyme intermediate to nucleoside diphosphates resulting in the synthesis of nucleoside triphosphates other than ATP [15,16,17] (Figure 2). But several additional enzymatic functions have been suggested for NME1, including serine-threonine kinase activity, geranyl and farnesyl pyrophosphate kinase activity, and a 3’-5’ exonuclease activity on DNA [11,18,19,20]. Interestingly, the NME1 histidine kinase activity depends upon catalytic transfer of the activated phosphate from the autophosphorylated histidine 118 residue (H118) onto a histidine in a target protein instead of onto a nucleoside diphosphate [21,22]. The highly conserved nature of this catalytic region and its histidine residue between species implies that this protein kinase activity remains central.

NME kinases are ubiquitous, but only a few NME partners and protein substrates are currently known. An additional complexity is that NME family members can interact with each other, raising the functional importance of NME oligomerization, that will be also addressed. 

Disregarding the molecular mechanism, NME members are reportedly involved in multiple physiological and pathological conditions [23]. Indeed, *NME1* was originally defined as the first metastasis suppressor gene, because its RNA level was higher in tumors with low metastatic potential [24]. Besides the downregulation in metastatic tumors, a dual regulation of NME1 during tumorigenesis was found, with overexpression being observed during early stages of primary tumor growth [25]. NME1 is also involved in membrane remodeling and nucleotide channeling [26], and knockout experiments targeting NME1 can cause growth deficiency while the double knockout of NME1/NME2 is lethal at birth in mice, and has impaired T cell activation [27,28].

Taken together, the publications related to NME and the synonymous NM23 or NDPK, have grown significantly since 1990, with around 1823 publications according to PubMed. Recent progress in elucidating the functions of this intriguing protein family was discussed by experts in the field at the 11th International Congress on the NDPK/NME/AWD protein family in October 2019 in Annecy, France. Their presentations clearly illustrated the complexity mentioned here, with emerging importance for understudied members such as NME3/4/6/7. In this review, we focus on the histidine kinase activity of the NME family, and its role in physiological and pathological conditions related to NME family members.

### 1.2. The Resurgence of Histidine Phophorylation

Phosphohistidine (pHis) belongs to the N-phosphate class of SONAtes (for S-O-N-A phosphate categories respectively, corresponding to thiophosphate, phosphate-ester, phosphoramidate, and acyl-phosphate bonds) and is characterized by an acid-labile and thermosensitive phosphoramidate bond, in contrast to the O-phosphate category, which includes the conventional phosphoserine (pSer), phosphothreonine (pThr) and phosphotyrosine (pTyr) [3,9,29]. pHis is also the only phosphoamino acid with two isomers; histidine can be phosphorylated in N1 (1-pHis or π-pHis) or N3 (3-pHis or τ-pHis) position nitrogen atoms in the imidazole ring. 

Histidine phosphorylation is particularly well-known in the two-component system (TCS) or multi-component phosphorylation system used in bacteria for cell signal transduction. In TCS signaling, a transmembrane histidine kinase, generally a sensor kinase, is autophosphorylated on a catalytic histidine, and this phosphate is then transferred onto an aspartate in a response regulator protein, which transmits the signal. Histidine phosphorylation is found not only in bacteria, but also in various other organisms such as fungi and plants and more recently has been associated with important mammalian cellular processes [30,31].

Despite its long history, the mammalian pHis proteome has been difficult to study because of the acid-lability and thermosensitivity of this modification, which is not compatible with most of the standard phosphoproteomic methods used for pSer, pThr and pTyr analysis. Indeed, this is one of the reasons why our knowledge about phosphorylating events that generate phosphoramidate bonds has progressed slowly. The higher free energy of these P-N bonds leads to rapid hydrolysis at low pH or upon exposure to high heat. Even exposure to mild heat and acidic conditions, such as pH = 6.0 and 60 °C for 30 min, as well as certain primary amines, effectively hydrolyzes pHis. It has also been reported that the 1-pHis isomer is less stable than the 3-pHis isomer [32,33]. 

Yet, over the last few years, several new tools have emerged and have led to a resurgence in the study of histidine phosphorylation, specifically thanks to the development of histidine phosphate analogs and pHis monoclonal antibodies [34,35,36,37]. In parallel, diverse methods were developed for the enrichment or detection of pHis using immunobinding [38,39,40,41,42,43], autoradiography [21,44], and NMR or mass spectrometry [22,45,46,47,48,49]. 

These recent studies have begun to highlight the potential of histidine phosphorylation in multicellular organisms as a reversible regulatory protein modification and support a possible role for histidine kinases in cell signaling. 

### 1.3. The Pros and Cons of Histidine Kinase Potential of NME

The intrinsic nucleoside diphosphate kinase (NDPK) activity associated with the *NM23* gene products, primarily NME1-4, is seemingly not necessary for all of their biological functions [50]. This strongly suggests that some of these functions could be related to its unexplored histidine kinase activity.

Additionally, studies in *E. coli* showed that NDP kinase can phosphorylate histidine protein kinases, such as EnvZ and CheA, which both catalyze pHis-pAsp phosphorelay signal transduction systems in bacterial TCS [51]. Similarly, NME1 histidine kinase activity was associated with the motility suppressive activity of NME1 in breast carcinoma [52,53]. Additionally, based on eukaryotic two-component pathway homologues found in *Arabidopsis thaliana*, one report suggested that NME1 phosphorylated the kinase suppressor of Ras (KSR) at Ser392, as well as Ser434 when Ser392 was mutated [54].

Several studies have shown that NME phosphorylates the histidine in the catalytic site of ATP-citrate lyase (ACLY) [55,56,57] and can transfer a phosphate from its catalytic histidine to an aspartic residue, identified as Asp-319 on aldolase C, when using a wild type NME1 but not a P96S or S120G mutant NME1 [58].

Interestingly, NME2 forms complexes with βγ subunits of heterotrimeric G-proteins and contributes to G protein activation by increasing the high-energy phosphate transfer onto GDP via formation of a phospho-H266 intermediate in Gβ subunits [17]. Furthermore, the use of NME2 or a catalytically inactive H118N-NME2 in a combined NME1 and NME2 null background, revealed that the NME2-dependent phosphotransfer reaction requiring its catalytic activity, specifically allowed receptor-independent basal G(s) activation [59]. NME2 is also implicated in phosphorylation of H358 of KCa3.1 potassium channel leading to its activation by relief of copper inhibition [60,61].

These diverse observations are among several types of evidence that support the concept that NME acts as a histidine kinase. However, as will be discussed further, one issue with NME acting as a protein kinase is that structure of its active site is designed to bind nucleotide tri/diphosphates, which might be expected to restrict access of protein histidine residues. Nevertheless, the fact that histidine residues such as H266 of Gβ are exposed on the protein surface, and also the recent finding that the NME1 activator molecule (NMac1) induces NDPK activation through allosteric conformational changes, suggest possible ways in which NMEs could use proteins as substrates [17,62].

Newly developed tools, such as sequence-dependent phospho-specific NME antibodies and NME inhibitors [9,63], will facilitate the study of histidine phosphorylation in numerous models, and combined with the growing interest in histidine phosphorylation, provide an impetus to explore the potential function of NMEs as protein kinases.

## 2. NDPK and Protein Kinase Activity: from Structure to Function

### 2.1. General Architecture of Human NMEs

**Figure 3:**
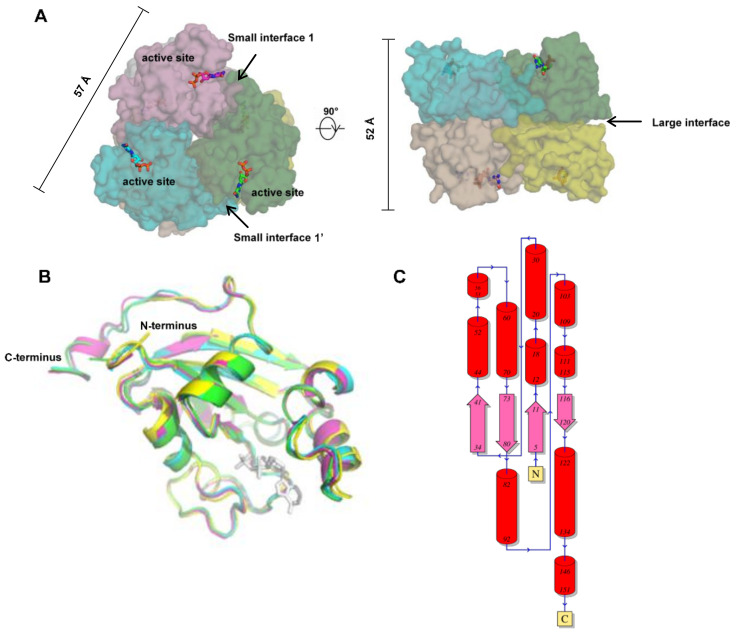
Structures of human NMEs. (**A**) Human NMEs 1-4 are hexamers with a D3 symmetry. Each subunit contains an active site, as represented with sticks. The six subunits are shown in different colors. Each subunit forms three interfaces with adjacent subunits, as indicated by arrows. The diameter (shown in the top view, left panel) and width (shown in the side view, right panel) are 57 Å and 52 Å, respectively. (**B**) Superimposition of human NME1–4 subunits (colored green, cyan, yellow, and magenta, respectively). A nucleoside diphosphate (NDP) substrate is shown in white sticks. (**C**) The topology of human NMEs, with helices represented by cylinders and strands by wide arrows. The topology of human NME1 is used as a representative (PDB ID: 1UCN, figure from PDBsum).

During the past 25 years, more than 10 structures of human NMEs have been solved, including crystal structures of NME1, NME2, NME3, NME4, and NME10 (Table 1). The first reported structure was for NME2 (also named NM23-H2, PDB ID: 1NSK) at 2.8 Å resolution [64]. The structure demonstrated that human NME2 adopts a hexameric architecture, i.e., a dimer of trimers or a trimer of dimers, with a D3 symmetry (Figure 3A). The molecular weight of each monomer is about 17 kDa, and the hexamer is ~100kDa. The monomers fold into a compact α/β domain, followed by a C-terminal extension (Figure 3B). The α/β domain is built around an anti-parallel β sheet with β4β1β3β2 topology, surrounded by six helices (Figure 3C). Each monomer contains an active site, located in the cleft against the adjacent subunit (Figure 3A). 

Human NME1-4 are highly conserved (sequence identity > 50%), but relatively distinct from human NME5–10. Since 2002, structures of other human NME subtypes have been solved, showing highly similar architectures compared to NME2 (Figure 3). NME1, 2, 3, and 4 all adopt hexamer conformations with D3 symmetry. In addition, all of them demonstrate a similar topology (Figure 3C), with RMSDs lower than 1.2 Å (calculated by align with Cα). Despite highly conserved sequences and structures, the cellular functions of human NME1-4 are distinct as mentioned in the previous section. 

### 2.2. Structural Basics of the Catalysis of Human NMEs

Human NMEs have been found to be multifunctional enzymes, with activities including NDP kinase, protein kinase, and 3′-5′ exonuclease. Substrates of the NMEs acting as NDP kinases and 3′-5′ exonuclease (NDP and dinucleotide respectively) have been observed bound to the same site of the enzyme (PDB ID: 3BBB [70]). Despite the many substrate-bound structures of NMEs, mostly with nucleotides, no structures have been reported with a bound protein substrate, but functional studies suggested that the phosphotransfer step takes place via the same active site, also through transfer of phosphate from H118 [66].

**Figure 4:**
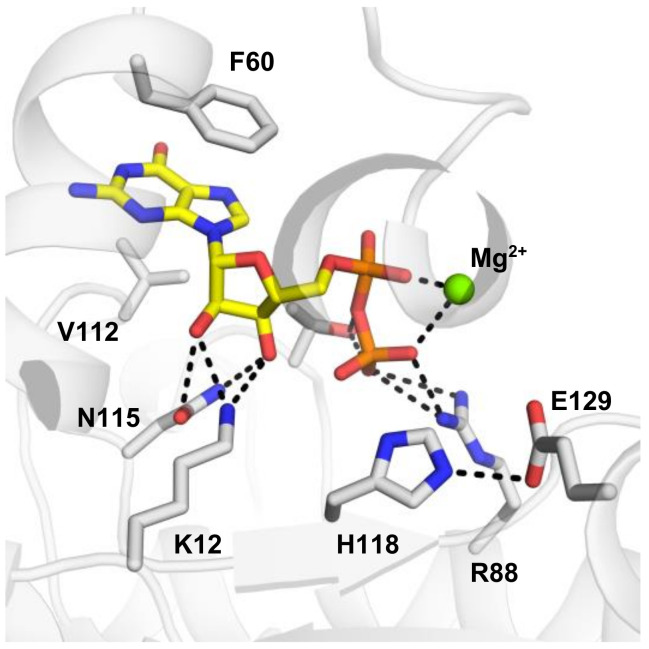
Active site of human NME2. Human NME2 is shown in light gray, with residues essential for substrate binding are highlighted in sticks. Complexed substrate Guanosine diphosphate (GDP) is shown in yellow with the β-phosphate lying close to the τ/N1 position of H118. Hydrogen bonds are shown in black dashed lines.

Crystal structures of apo-form and substrate-complexed forms of NDP kinase have been determined for several different species, e.g., *H. sapiens*, *M. xanthus,* and *D. discoideum*, etc. Both ADP and GDP substrates bind to the NDP kinases in a similar manner. The active site is located in a cleft formed by two helices (helix 44–52 and helix 60–70). The base of the nucleotide is near the protein surface, exposed to the solvent. More specifically, by using NME2 as an example, F60 of helix 60–70 and V112 of the Kpn loop (94 to 114) form a clamp, stabilizing the base of the substrate (Figure 4). Compared to the apo structure, the helix 44–52, the helix 60–70 and the turn in between move about 1 Å toward the substrate, facilitating a Pi-Pi bond interaction between F60 and the base ring of the nucleotide. The base remains largely accessible to the solvent. Intriguingly, the N2 amino group of the substrate GDP forms a salt bridge to E152 of the adjacent subunit in human NME2 (PDB:1NUE, a human NME2 structure complexed with GDP). However, there is no such interaction between ADP and the adjacent subunit (shown in PDB: 1UCN, a human NME1 structure complexed with ADP). The E152 residue is conserved between human NME1 and NME2. The clamp residues, F60 and V112 are conserved between NME1-NME3, and for NME4, the V112 is replaced by an isoleucine that would presumably retain the interaction, corresponding to the observation that NME1 to NME4 all possess NDPK activity. On the contrary, the other NMEs (NME5-NME10) are not able to form the clamp due to the absence of a residue equivalent to V112 (Figure 1B).

In contrast to the base, the ribose is buried in the protein. Side chains of K12 and N115 form hydrogen bonds to the 2′ and 3′ hydroxyls groups. The pyro-phosphate moiety points towards H118. T94, and R88, which form hydrogen bonds with the phosphogroups of the substrate. H118 is responsible for phosphotransferase of the τ-phosphate and is stabilized by E129 in the state shown in Figure 4. Crystal structures of NMEs showed that the N3 position of H118 forms hydrogen bonds to E129, leaving the N1 position available to be phosphorylated and responsible for subsequent phosphotransfer. In all the human NMEs with NDPK activity (group I cluster from phylogenetic analysis [72]: NME1-4), all of these residues, T94 and R88 are conserved. However, for the other NMEs, not all these residues are conserved (Figure 1B) which may contribute to their weak or complete lack of NDPK activity. 

NME1 and NME2 have been demonstrated to act as protein kinases using pH118 as an intermediate activated state for phosphotransfer to substrate proteins. H118 is conserved between NME1 and NME2. NME3/4/5/6/8 also have a histidine at the same position, but while NME3 and 4 possess NDPK activity, none of them have been shown to be able to phosphorylate protein substrates. Further studies need to be carried out to elucidate their activities and catalytic mechanisms. 

It has been shown that human NME1/5/7/8 have the ability to act as 3′–5′ exonucleases [73]. K12, Q17, R88, and R105 were suggested to be critical for DNA binding and cleavage. Q17 is only required for DNA cleavage but not for its NDPK activity. On the contrary, Y52, N115, H118 were found to be essential only for the NDPK activity [74]. 

### 2.3. Assembly and Oligomerization of Human NMEs

Human NMEs form hexamers, as is the case for rat and bovine retina, whereas *Myxococcus* NME is a tetramer, although all their topologies are essentially identical [75]. Dimers and monomers have also been found by size exclusion chromatography in some cases [64,76,77]. Different oligomerization states of NME1/2 might bias its phosphotransferase activity either towards NDP phosphorylation or protein-histidine phosphorylation.

To form a hexamer with D3 symmetry, each monomer forms three different interfaces with adjacent subunits, including a large interface, a small interface close to the active site, and another small interface opposite the active site (Figure 3A). Interface areas and the Gibbs free energy of each interface in human NME1 to 4 have been calculated using the Proteins, Interfaces, Structures, and Assemblies (PISA) software (shown in Table 2) [78]. For the large interfaces, the areas are approximately 1000 Å^2^, whereas for both small interfaces of each NME the areas are approximately 800 Å^2^. Amino acid residues forming the interface interactions are highlighted in yellow, cyan, and green for the large interface, the small interface 1 and the small interface 1′ respectively (Figure 3A). The interface amino acids are generally conserved between NME1-4, despite some variations. For instance, the C-terminal regions are responsible for a large portion of the small interface 1′. In NME1-3, this region is conserved but the C-terminal region is distinct in NME4. In the only available NME4 structure (PDB ID: 1EHW), the C-terminal region was not resolved, suggesting that it is highly flexible in solution. This unsolved region is shown in italics in Figure 1B. Due to the lack of the C-terminal region in the solved structure, the calculated area of the human NME4 small interface may only occupy a portion of the real interface area. Distinct from NME1-4, the interface amino acids of other human NMEs are not conserved, implying that they may have different assembly modes. Intriguingly, it has previously been noted that only one of the interfaces (i.e., the large interface) is conserved between human NME2 and *M. xanthus* NDP kinase [64], where the latter assembles as a tetramer. This observation suggests that evolution of one or two of the multiple interfaces may result in a different assembly and thereby allow adaptation to different functions in various species. 

Under oxidizing conditions, disulfide bond formation leads to a dissociation of the hexameric structure, which consequently increases the dimeric population and reduces activity of NME1 [79]. This structure has been solved (PDB 4ENO) and shows an intramolecular Cys4 and Cys145 disulfide bond, which causes a conformational change in the C-terminal region and disrupts the small interface, thereby destabilizing the hexamer state and triggering dissociation [67]. NME1 was also shown to have physiologic relevance in the oxidative stress response. NME1 null mice exhibit lower tolerance to oxidative stress compared with wild-type (WT) mice [80]. This function may be regulated by redox-dependent oligomerization. 

Furthermore, given the importance of heterooligomerization in the physiological activities of NME1, 2, and 3, NME family members could exert their activities as heteromeric complexes or even single subunits [81]. Because of their nearly identical sequence, NME1 and NME2 are able to form heterohexameric isoenzymes as well as homo-hexamers [82], even though 18 out of 152 amino acids are different, such that NME1 is an acidic protein, whereas NME2 is a basic protein. The study of NME1-NME2 interaction in vivo by the use of FRET/FLIM revealed that NME1 and NME2 co-localize, with irradiation-induced DNA damage causing a small shift in NME1/NME2 homo- and hetero-isoenzyme ratios [83]. In agreement with this, it has been observed more recently that NME3 stimulates mitochondrial elongation dependent on oligomerization activity [84]. Additionally, previous observations based on atypical modes of signal transduction in bacteria, show that inactivation of histidine kinase activity can occur via homo- or hetero-oligomerization and cross-phosphorylation between histidine kinases in TCS and a similar process could be involved in more evolved histidine kinases such as NMEs [85].

The differences in quaternary structure between species, the assembly into homo/hetero isoenzymes, and the oxidation-induced NME dimer formation all suggest that oligomerization can be a potential mode of regulating NME multifunctionality.

## 3. NME as a Protein Histidine Kinase 

### 3.1. NME Binding Partners

Multiple biological functions of NME have been described and reflect on its numerous binding partners, even though only some of them have been identified [86]. Among the identified NME protein substrates that are currently known to be histidine phosphorylated (Table 3), the calcium channel TRPV5 is phosphorylated on H711 (3-pHis) regulating Ca2+ reabsorption [87], GNB1 on H266 (3-pHis) facilitating GPCR signal transduction [17], the calcium-activated potassium channel KCa3.1 (also known as SK4) on H358 (3-pHis) activating KCa3.1 channel opening by antagonizing copper-mediated inhibition [60,61], the ATP-citrate synthase ACLY on H760 (3-pHis) [56], and succinate thiokinase (SUCLG1) or succinyl-CoA synthetase on H299 (3-pHis) [52,53,88]. K-Ras suppressor (KSR1) [54] and annexin I [89] are also reported substrates of NME, with KSR1 being phosphorylated on serine and annexin I on histidine [30,90]. 

In a TCS-like manner, NME1 transfers its histidine phosphate to D319 of aldolase C [58]. Interestingly, NME1 directly enhances the expression of aldolase C by binding to its promoter [91]. Moreover, using a bioinformatics approach, aldolase C has been identified as one of the genes establishing the NME1-regulated Metastasis Suppressor Signature (MSS) [92]. It would be of interest to see if other genes that make up the MSS are also phosphorylated by NME on histidine or aspartate. The sequences around phosphorylated histidines in ACLY and SUCLG1 resemble the sequence surrounding the phosphorylated aspartate in aldolase C, all containing an AGA sequence towards the C-terminal side of the phosphorylated site [58]. Until now, no histidine phosphorylation sequence motif has been identified despite some recent phosphoproteomic analysis efforts and attempts [93], although leucine and isoleucine are overrepresented in sequences surrounding phosphorylated histidine, lysine, and arginine [9,94,95]. 

Apart from its substrates, several proteins are known to interact with NME members as identified by different in vitro methods [86]. These binding partners include Tiam1, which is negatively regulated by NME1 and results in the reduced activation of Rac1, a regulator of cell motility [96]. NME1 also binds to Db1-1 a specific exchange factor of the Rho-GTPase CDC42 [97] to inhibit cell migration [97]. Similarly, NME1 binds to gelsolin and inactivates its effects on actin to suppress tumor motility [98]. In breast cancer cell lines, NME1 interacts with h-Prune [99], as is the case in the fruit fly *Drosophila melanogaster*. The NME1/hPrune complex is induced by the phosphorylation of NME1 S122 and S125 by CK1 [100]. Whether phosphorylated histidine residues regulate the formation of this complex is still unknown. Studies in *Drosophila* [26] and mammalian cell lines demonstrate that NME interacts with dynamin 1 to regulate cell migration through the internalization of chemotactic receptors in migrating cells [101,102,103]. Although it is proposed that NME acts through its NDPK function to generate GTP locally for dynamin GTPase function, whether its histidine protein kinase function also plays an important role in this interaction is unknown. In general, it is still undefined if NME acts through its protein kinase activity and phosphorylates histidine residues in these binding partners to regulate cell migration and invasion. 

NME kinases are ubiquitous, but their activities and substrates are associated with diverse subcellular (cytosol, mitochondria, and nucleus) and even extracellular localizations [73,104,105,106]. Besides specific subcellular localization, one of the challenges is to define if these substrates are isoform-specific or whether they are common and redundant to several NME members, as well as how far the list of interacting partners diverges between isoforms and defines individual functions [107]. Most of the known substrates have been based on NME1 or 2 activities only, but considering the autophosphorylation of NME1 and 2 on H118 (1-pHis), some of the NME kinases could also be considered as common direct substrates. It is still uncertain if there is any specific substrate for each of the NME members, which could be used to monitor activity. For instance, research with the mitochondrially located NME4 show that it binds selectively to the lipid cardiolipin [108], is associated with SUCLG1 [109], interacts with gamma-aminobutyrate transaminase (ABAT) [110], the dynamin-related GTPase OPA1 [111], and is known in *Xenopus* to bind p27Xic1 a cyclin-dependent kinase inhibitor [112]. Further research on interacting partners of other NME members will help elucidate their cellular functions and if its histidine kinase function plays a role on them.

### 3.2. NME Protein Histidine Kinase Functions as Revealed by Mutagenesis

To understand the biological effects related to NME’s function as a protein-histidine kinase, site-directed mutagenesis of the catalytic site has been performed. Mutations of H118 to prevent its autophosphorylation, which is essential for NME kinase function, have been explored in several models. In *Drosophila*, the equivalent histidine is H119 in the *awd* gene, which encodes the NME homolog. Loss of function of the *awd* gene can be rescued by expression of WT AWD but not with the mutated H119A form, showing the importance of the catalytic site in this gene function [113]. On an *NME1/2* null background, re-expression of NME2 H118N catalytic inactive mutant led to lower basal G protein activation compared to the WT form [59]. This reveals the importance of NME2-mediated histidine phosphorylation of the G beta subunit for cAMP production by G protein activation. 

Because H118 is important for both nucleotide and protein-histidine kinase activity, experiments carried out by mutating only this amino acid are unable to define which activity is responsible for the observed phenotypes. To distinguish between these two functions, the consequences of mutating Pro96 of NME1 has been investigated. Mutation of Pro96 to serine causes conformational changes in NME affecting its binding to protein substrates [114]. This mutation was first identified in *Drosophila* as the “killer of prune” mutation since it was lethal in combination with the *prune* gene mutation [115,116,117]. Therefore, mutation of H118 abrogates both nucleotide and protein kinase activity, and mutation of Pro96 compromises only its protein kinase activity [53]. Recent findings indicate that NME1′s ability to suppress motility depends on its protein kinase function, as neither mutant is able to inhibit migration of transfected MDA-MB-231 and MDA-MB-435 breast cancer cells [114]. Interestingly, migrating cells overexpressing NME1 had lower levels of its 1-pHis form compared to stationary cells. These data suggest a role of 1-pHis phosphorylation and phosphorylated NME (pNME) in cell migration. 

**Figure 5:**
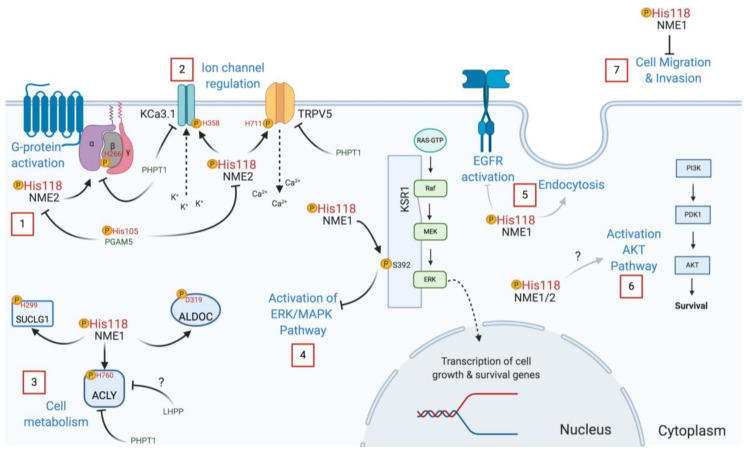
NME protein-histidine kinase functions. 1. NME regulates G-protein activation through histidine phosphorylation of the G-protein beta subunit 1 (H266). 2. NME modulates ion channel regulation via histidine phosphorylation of the potassium channel KCa3.1 (H358) and the calcium channel TRPV5 (H711). 3. NME also plays a role in cell metabolism by phosphorylating ACLY (H760) to promote fatty acid biosynthesis, SUCLG1 (H299) for succinyl CoA production and ALDOC an important enzyme in glycolysis. 4. NME inhibits the activation of the ERK/MAPK pathway by the phosphorylation of KSR (S392). 5. NME protein kinase has been implicated in EGFR activation and endocytosis. 6. pNME1/2 levels might play a role in activation of the AKT pathway. 7. NME phosphorylation inhibits cell migration and invasion in cancer cell lines. Histidine phosphatases (green), such as PHPT1, dephosphorylate some of the substrates of NME, and the PGAM5 (H105) histidine phosphatase dephosphorylates NME2 itself. No direct substrates have been confirmed for LHPP, another histidine phosphatase, even though ACLY has been suggested as a candidate. This image was created with Biorender.com.

Another recent paper from the Steeg group reported that P96S mutant NME1 was unable to promote endocytosis or inhibit EGFR activation (Figure 5), demonstrating that these effects are mostly due to its protein-histidine kinase activity [102]. A simultaneous study revealed that NME1 interacts directly with the calcium-calmodulin-dependent kinase II (CAMKII). However, the H118 catalytic site mutant was as effective as the WT form of the enzyme, implying that CAMKII regulation does not involve its phosphotransfer activity [118]. Moreover, human recombinant NME1 H118F and P96S mutants were unable to phosphorylate KSR, and in transfected HEK293T cells, the P96S mutant failed to suppress unlike WT NME1, suggesting that NME1 acts as a protein kinase transferring its histidine phosphate to a serine in the KSR pathway [54]. Additional work with the mutated P96S and H118F forms of NME1 showed that phosphorylation of aldolase C and SUCLG1 is compromised with these mutants [53,58]. The S120G mutation in NME1 has also been studied since it was found in neuroblastoma [119]. Interestingly, the S120G mutation is deficient in histidine and serine autophosphorylation, but only exhibits a slight decrease in its protein-histidine kinase function [53]. 

Work using NME mutants has revealed how its histidine kinase function is important in cell migration and invasion, in cellular growth and development, endocytosis, and cell signal transduction. As a summary, some NME protein kinase functions are depicted in Figure 5. Further studies are needed to elucidate how histidine phosphorylation regulates other biological processes. 

### 3.3. Histidine Phosphatases

Histidine phosphorylation can be reversed by phosphohistidine phosphatases, but as is the case for histidine kinases, only a few are currently known. NME2 pH118 is dephosphorylated by Phosphoglycerate Mutase Family Member 5 (PGAM5) (Figure 5). PGAM5 has also been reported to be a pSer phosphatase; in both cases, the PGAM5 catalytic H105 attacks the phosphate linkage in the substrate forming a pH105 intermediate that is then hydrolyzed releasing phosphate [120]. Phosphohistidine Phosphatase 1 (PHPT1) was the first histidine phosphatase discovered, back in 1962 [3]. PHPT1 acts on many of the same proteins that are phosphorylated by NME1/2, including KCa3.1 [121], TRPV5 [87], ACLY [57], and GNB1 [122,123]. In this way, NME and PHPT1 modulate the activity of these proteins by reversible histidine phosphorylation (Figure 5). Interestingly, PHPT1 knockout mice exhibit neonatal hypoglycemia due to the abrogated trafficking of KATP channels in pancreatic beta cells, a condition similar to congenital hyperinsulinism [124].

Another recently discovered histidine phosphatase is phospholysine phosphohistidine inorganic pyrophosphate phosphatase (LHPP), which was first purified from swine brain tissue [125]. Although, as its name indicates, LHPP. is not selective for histidine dephosphorylation. LHPP. acts as a tumor suppressor in liver cancer where its expression is downregulated, whereas pNME1/2 are upregulated, resulting in increased histidine phosphorylation of proteins [126]. Other studies in colorectal [127], bladder [128], pancreatic [129], thyroid [130] and cervical cancer [131] have demonstrated that LHPP. is downregulated in cancer [132]. Based on GWAS studies, LHPP. has also been implicated in major depressive disorders [133,134,135], alcohol dependence, and risky behavior [136]. A confirmed substrate for LHPP. has not been identified although based on mass spectrometry analysis of pHis-containing proteins from mouse hepatocellular carcinoma cells ACLY has been suggested to be one [126]. However, even though LHPP. can dephosphorylate low molecular weight compounds with P-N bonds, such as pHis, structural analysis of the LHPP. catalytic pocket suggests that pHis residues in proteins might be sterically restricted from gaining access to the active site ([137]; R. Kaligiri, personal communication), in one sense analogous to the issue of how protein histidines gain access to the active site of NME. The molecular mechanism of LHPP. action and the role of histidine phosphorylation in other types of cancers is largely unexplored, but evidence from ongoing research in our group suggests that it might be tissue type and cell-context dependent, and might play an important role. LHPP, PGAM5, and PHPT1 are all evolutionarily, conserved highlighting the important function of these proteins. 

Other phosphatases such as STS-1 and STS-2 are members of the PGAM family and share sequences with histidine phosphatases. Therefore, they are potential candidates to dephosphorylate histidine residues possibly targeted by the NME histidine kinase [138]. Other phosphatases that are not specific for pHis also can dephosphorylate this phosphoamino acid. These include PP1, PP2A, and PP2C. However, no further evidence has been reported in recent years regarding the histidine phosphatase function of PP1, PP2A, or PP2C towards known pHis protein substrates besides histone H4. The mechanisms underlying histidine phosphatase substrate selection are poorly understood and the main factors driving their substrate specificity are unknown. A non-redundant role for histidine phosphatases to regulate different targets in cell signaling pathways has been suggested [120]. For these reasons, it is important to continue expanding our understanding of histidine phosphatases in parallel with investigating histidine kinases, such as NME. The understanding of these understudied enzymes and the role of histidine phosphorylation in eukaryotes might point to new therapeutic targets for diseases. 

## 4. Role of NME Protein Histidine Phosphorylation Function in Cell Signaling

While many of the molecular functions of NME in the cell remain unknown, recent progress has been made by investigating NME binding partners. To a large extent, NME function and its partners have been studied in the context of cancer, an outgrowth of the fact that NME was first described as a metastasis suppressor gene. Nevertheless, studies in different organisms have revealed that NME is essential for biological activities related to development and other disorders in addition to cancer. Not a lot is known about NME protein kinase activity and cell signaling. Here, we discuss the few instances where it is known that NME regulates signal transduction through histidine phosphorylation. However, most of the following studies relate to NME function and signaling modulation in different models, independent of its protein histidine kinase activity, but it is interesting to reconsider some of them in the light of histidine phosphorylation. 

### 4.1. NME Histidine Phosphorylation Signaling in Tumor Metastasis Suppression 

NME1 was first described as a metastasis suppressor in a study comparing RNA levels in mouse metastatic melanoma cell lines with non-metastatic cells, which showed that Nm23/NME1 expression was downregulated in the metastatic cells [24]. NME1 is overexpressed in the primary tumor in the early tumorigenesis but lost during metastasis [139]. In melanomas and epithelial tumors, NME1 expression is inversely correlated with metastasis [25,92,111,140,141,142,143]. In contrast, in hematological malignancies and other types of cancer, such as ovarian, prostate, and neuroblastoma, higher expression of NME1 is correlated with a bad prognostic [144,145,146,147]. These contradictory data might be explained by cell-type differences in NME-modulated signaling pathways.

NME has a cell proliferation inhibitory function. To achieve this, NME1 through its protein phosphotransfer function activates KSR by phosphorylation of S392 leading to altered subcellular localization [54]. KSR is important for the assembly of the Raf/MEK/ERK complex and activation of components of ERK/MAPK signaling pathway [148,149,150]. Similarly, in breast carcinoma cells NME1 overexpression attenuates activation of ERK [139]. Meanwhile, silencing of NME1 in HepG2 cells results in hyperactivation of ERK [139]. This interaction has also been confirmed in *Caenorhabditis elegans* Masoudi, et al. [151]. Deletion of the NME1 homolog NDK-1 leads to a protruding vulva (Pvl) phenotype, in which EGFR/Ras/MAPK signaling plays a role [151,152]. pMAPK levels are reduced in these mutants [151]. Surprisingly, unlike in humans, NDK-1 activates the Ras/MAPK signaling pathway [54,153]. A possible explanation of this difference is the differential regulation of the KSR scaffold [103]. Nevertheless, these data suggest that NME1 regulates Ras/MAPK signaling through its protein kinase function.

Another signaling pathway where histidine phosphorylated NME might act is through the AKT signaling pathway. In a mouse hepatocellular carcinoma model, levels of activated pS473 AKT were increased in tumors expressing higher levels of NME and its histidine phosphorylated form compared to the normal control [126]. Although silencing of NME1 in HepG2 and HCT8/S11 cells resulted in increased pAKT levels [139]. Despite this possibility, no study has confirmed the direct impact of NME histidine phosphorylation on this signaling pathway. An additional possible mechanism by which NME acts as a metastasis suppressor is through the histidine phosphorylation of ACLY. This protein is an important intermediate in the formation of acetyl CoA from citrate, which is correlated with pancreatic cancer progression, as tumors fuel their growth through fatty acid synthesis [154,155]. Therefore, NME might regulate tumor growth through modulation of anabolic pathways. 

There are other important signaling pathways regulated by NME for metastasis inhibition. However, there is no direct link between histidine phosphorylation and NME function in these cases, and it remains to be determined whether histidine phosphorylation might be important in some of them. For instance, silencing of NME increases Rac1 signaling and MAPKs, including ERK, JNK, and PI3K/AKT pathway activation [139]. NME1 also regulates cell motility through its interaction with Tiam1 and Db1-1, which reduces activation of Rho - Rac1 signaling [96,97]. The regulation of MLC phosphorylation by NME1 modulates its cell invasion function [156]. NME1 overexpression decreases MLC phosphorylation and decreases activation of the ERK MAPK pathway; however, which protein NME acts on in this pathway remains unknown. Moreover, TGF- β treatment of lung cancer cell lines deleted for NME1 potentiates the epithelial-mesenchymal transition [157]. Correspondingly, in metastatic medulloblastoma the activation of the PRUNE1 signaling pathway involves PRUNE1 binding to NME1 and TGF-β activation [158]. NME regulates cell proliferation through EDG2 inhibition, which results in high p38/ERK activation in the lung in a mouse model of pulmonary metastasis [159,160]. Furthermore, NME is downregulated and EDG2 upregulated during myeloid differentiation of human leukemia cells [161]. 

NME1 can also promote cell death or enhance the engulfing phagocytic capacity of cells [103]. In *C. elegans*, binding of microRNA mir-35 to NDK-1 modulates the activation of the MAPK pathway resulting in DNA damage-induced apoptosis of germ cells [162]. In a caspase-independent apoptotic pathway manner, human NME1 acts together with the DNase TREX1 during granzyme A mediated cell death. In this way, NME1 overexpressing cells treated with granzyme A are more sensitive to DNA damage [163,164]. In *C. elegans* and in humans NME promotes the engulfment of apoptotic cells through its interaction with Dynamin1 and 2 [103,165,166]. In the same study, this function was also seen in murine models where NME1 silenced bone marrow-derived macrophages cleared fewer apoptotic thymocytes [166]. 

Is it possible that NME regulates MAPK/AKT signaling mediated cell proliferation, motility, and apoptosis by its histidine kinase activity? Because most of the previous studies did not focus on detecting pHis protein signals, this question cannot yet be answered, and future experiments, including with other NME family members, will be needed. Interestingly, ongoing work presented at the 11th International Conference on the NME gene family (NME 2019 conference) has highlighted a critical function for NME3 in maintaining genome stability and a possible role in breast tumor metastasis [167]. Likewise, another study showed that NME4 is related to cancer [105]. Still, there is insufficient published literature about how other NME members besides NME1 and 2 regulate different signaling pathways. More mutagenesis studies on different NME members, such as the ones discussed previously [54,102], should help our understanding of this role. Whether NME acts by its protein-histidine kinase activity in these cases is still unknown, but future studies detecting pH118 NME1/2 levels under pHis-preserving conditions will facilitate elucidation of the possible involvement of histidine kinase signaling in metastasis suppression.

### 4.2. NME Histidine Phosphorylation Function in Development and other Biological Processes

NME1 and 2 and the catalytic H118 residue are highly conserved from yeast to humans [9,72]. NMEs have been shown to have important functions in animal development [168] and other essential biological activities. NME2 histidine phosphorylation of the KCa3.1 potassium channel is needed for channel activation and the production of cytokines by CD4+ T cells [61,120,121]. In CD4+ T cells and mast cells PI3K-C2β is required for NME2 phosphorylation of KCa3.1 [169,170]. PI3K-C2β signaling may be also required for NME2 phosphorylation of H711 TRPV5 and Ca^2+^ channel activity [87], which is important for calcium homeostasis regulation [171]. Phosphorylation of TRPV5 by NME2 might inactivate TRPV5 in a manner similar to the way in which calmodulin binding inhibits TRPV5 function [87]. Moreover, phosphorylation of H266 on the Gβ subunit by NME2 is a key intermediate in the activation of G protein signaling, needed for the regulation of cardiac contractility [17,172,173]. One study with NME3 determined that its catalytic activity is important for cell viability, and detected the 1-pHis form of NME3 under glucose starvation conditions [84]. The same study also suggested that that homozygous loss of NME3 function results in a fatal human neurodegenerative disorder.

Work with *Drosophila* has demonstrated that the deletion of the NME homolog *awd* gene, causes defects in the formation of imaginal discs during the development of larvae to adults [174]. In the fruit fly, *awd* plays a role in the migration of cells during tracheogenesis and border cell movement, with *awd* working through receptor endocytosis to modulate the activation of FGFR/VEGFR/PDGFR and JAK/STAT signaling [175]. In *C. elegans* NDK-1 plays an important role in gonadal development [152]. In zebrafish, NME2 knockdown results in reduced cardiac contractility and decreased formation of vessels [176]. In *Xenopus,* the NME4 homolog, NM23-X4, is essential for the development of the retina [112]. In mice, NME1 and NME2 single knockouts are viable [27,177], but double knockouts are stunted in embryonic growth and die prenatally [178]. Furthermore, NME1 knockout females present poor mammary duct maturation [179]. In mouse embryos, NME1/2 might play an important role in erythroid lineage development, as they present with anemia and defects in erythrocyte maturation [178]. Moreover, NME5 and NME7 knockout mice show phenotypes related to impaired cilia motility [180,181,182]. Conditional NME family knockouts targeting different tissue types will improve our understanding of NME function in specific contexts, as all the knockout studies so far are constitutive [103]. Furthermore, knock-in mice deficient for NME NDPK and protein-histidine kinase activities should be made to explore the role of protein-histidine phosphorylation in specific functions. 

Besides development and cancer, there are studies showing the importance of NME in other biological contexts. Work with *awd* and *shibire* (dynamin homolog) in the *Drosophila* demonstrates that *awd* functions as a GTP supplier to dynamin to mediate endocytosis [26]. This has also been studied in human and monkey cell lines, where NME plays a role in the endocytosis of several receptors through this interaction to inhibit cell motility [101,102]. NME1/2 is also important in other diseases such as cystic fibrosis through its interaction with CFTR [183,184]. Moreover, NME2 might also play a role in glucose metabolism [185], and NME1/Prune complex could be related to neurodevelopment disorders [186]. All these roles of NME1/2 in different biological processes underscore the importance of unveiling the role of histidine phosphorylation in development and in common diseases.

### 4.3. Detecting Histidine Phosphorylated NME in the Cell

The detection of histidine phosphorylated NME has been challenging given that, until five years ago, we lacked the appropriate tools to selectively detect 1-pHis and 3-pHis in proteins. This is mainly why most of the work done so far has not explored NME protein-histidine kinase activity by evaluating changes in pHis protein levels. This began to change with the development of stable phosphoryltriazolylalanine (Tza) analogs of 1-pHis and 3-pHis by the Muir and Webb groups [35,187], which allowed the development of antibodies selective for the two isoforms of pHis. Kee, Villani, Carpenter and Muir [35] were the first to develop a 3-pHis antibody against histone H4 pH18. The same group later developed sequence-independent antibodies towards 3-pHis [36,188] and McAllister, et al. [189] designed additional triazole phosphohistidine analogs that later were used to generate antibodies to detect pHis. However, the first-generation antibodies produced against these 3-pHis analogs had cross-reactivity with pTyr. Our group used 1-pHis and 3-pHis Tza analogs to develop sequence-independent monoclonal antibodies to detect 1-pHis and 3-pHis levels by immunoblotting, immunofluorescence, and enrichment for phosphoproteomics analysis with no cross-reactivity with pTyr [37]. With the development of 1-pHis monoclonal antibodies (mAbs), it was possible to detect pNME1/2 levels by immunoblotting with 1-pHis mAbs, which identified prominent 1-pHis-positive 17 kDa bands corresponding to NME1/2. One of these mAbs has been already used to detect elevated levels of pNME1/2 in hepatocellular carcinoma [126]. More recently, we have developed the first sequence-dependent anti-pH118 NME1/2 polyclonal antibody. By using these antibodies and the 1-pHis mAb, it has been possible to detect pNME H118 levels in HeLa and ALVA-31 prostate cancer cells [9]. Additionally, pNME1/2 could be specifically detected in a plethora of neuroblastoma [132], breast, and pancreatic cancer cell lines by our group. Ongoing work in the Hunter lab has also detected pNME in mouse breast and pancreatic tumors and normal tissue, although the pNME signal is lower than in cultured cells. It still remains unclear whether high levels of pH118 NME1/2 reflect increased NDPK or protein kinase catalytic activity. Instead, it could mean that NME1/2 are frozen in the activated state and are unable to transfer the phosphate. Such tools will aid the future validation and study of histidine phosphorylated NME in various models. 

The development of sequence-specific and sensitive monoclonal antibodies to individual pHis-containing proteins, and pH118 NME1/2 in particular, will be important for our understanding of the significance of NME protein histidine kinase activity in diverse cellular activities. There is a need for additional molecular tools in this field of study and complementary approaches, such as the development of selective NME histidine kinase inhibitors, would be valuable. Given the rapidly increasing amount of data and the identification of new histidine phosphorylated proteins, there is no doubt anymore that the extent of histidine phosphorylation was underestimated for many years but is now being recognized as an important regulatory post-translational modification. Many of the signaling roles of histidine phosphorylation remain to be explored, and important questions include whether other undefined histidine kinases and phosphatases exist and in what ways the addition of a phosphate to a histidine residue can initiate downstream signaling.

## Figures and Tables

**Table 1:** Available structures in PDB of all human NME1 to NME4 *.

PDB		Resolution	Ligand
NME1	1JXV [65]	2.2	apo
NME1	1UCN [66]	2	complexed with ADP
NME1	2HVD [16]	2.15	complexed with ADP
NME1	2HVE [16]	2.4	S120G, complexed with ADP
NME1	3L7U [64]	2.1	apo
NME1	4ENO [67]	2.8	under oxidative conditions trigger allostery
NME1	5UI4 [68]	2.75	conjugated to imidazole fluorosulfate
NME2	1NSK [64]	2.8	apo
NME2	1NUE [69]	2	complexed with GDP
NME2	3BBB [70]	1.3	complexed with dinucleotide d(AG)
NME2	3BBC [70]	1.7	R88A
NME2	3BBF [70]	1.7	complexed with GDP
NME3	1ZS6	2.3	complexed with ADP
NME4	1EHW [71]	2.4	apo

* The NME10 (XRP2) structure (PDB IDs: 2BX6, 3BH6 and 3BH7) were not included in this table as it only shares 22% identity in the NDPK domain and lacks important catalytic residues and NDP kinase activity.

**Table 2:** Interface area and Gibbs free energy of subunit interfaces of human NMEs.

	Large Interface		Small Interface 1 (Opposite to Active site)		Small Interface 1′ (on the Site of Active Site)		PDB ID
	Interface Area (Å^2^)	∆G (kcal/mol)	Interface Area (Å^2^)	∆G (kcal/mol)	Interface Area (Å^2^)	∆G (kcal/mol)	
NME1	1015	−15.3	808	−6.2	798	−6.7	1UCN
NME2	1015	−12.6	793	−6.7	780	−6.7	1NUE
NME3	1220	−11.5	836	−5.8	829	−4.3	1ZS6
NME4	998	−15.6	520	−1.4	509	−0.7	1EHW

**Table 3:** Known NME protein substrates.

Gene	Uniprot ID	Name	pHis Site	pHis Sequence	N1 or N3
**NME1**	P15531	Nucleoside diphosphate kinase A	H118	NII**H**GSD	1-pHis
**NME2**	P22392	Nucleoside diphosphate kinase B	H118	NII**H**GSD	1-pHis
**TRPV5**	Q9NQA5	Transient receptor potential cation channel subfamily V member 5	H711	TLG**H**LNL	3-pHis
**GNB1**	P62873	Guanine nucleotide-binding protein G(I)/G(S)/G(T) subunit beta-1	H266	TYS**H**DNI	3-pHis
**KCa3.1**	O15554	Intermediate conductance calcium-activated potassium channel protein 4	H358	RLK**H**RKL	3-pHis
**ACLY**	P53396	ATP-citrate synthase	H760	QFG**H**AGA	3-pHis
**SUCLG1**	P53597	Succinyl-CoA synthetase	H299	RMG**H**AGA	3-pHis
**KSR1**	Q8IVT5	Kinase suppressor of Ras 1	S392	RTE**S**VPS	-
**ANXA1**	P04083	Annexin A1	H246/H293?	YSK**H**DMN/	ND
				GTR**H**KAL	
**ALDOC**	P09972	Aldolase C	D319	GQR**D**NAG	-

## Abbreviations

NDPK	Nucleoside diphosphate kinase
NTPs	Nucleoside triphosphates
NDPs	Nucleoside diphosphates
PDB	Protein Data Bank
TCS	Two-component system
KSR	Kinase suppressor of Ras
MSS	Metastasis Suppressor Signature
MLC	Myosin light chain
pNME	Phosphorylated NME
GDP	Guanosine diphosphate
CAMKII	Calcium/Calmodulin-dependent kinase II
TRPV5	Transient receptor potential cation channel subfamily V member 5
GNB1	Guanine nucleotide-binding protein G(I)/G(S)/G(T) subunit beta-1
KCa3.1	Intermediate conductance calcium-activated potassium channel protein 4
ACLY	ATP-citrate synthase
SUCLG1	Succinyl-CoA synthetase
KSR1	Kinase suppressor of Ras 1
ANXA1	Annexin A1
ALDOC	Aldolase C
PGAM5	PGAM Family Member 5
PHPT1	Phosphohistidine Phosphatase 1
LHPP	Phospholysine phosphohistidine inorganic pyrophosphate phosphatase
GWAS	Genome-wide association studies
